# Non contiguous-finished genome sequence and description of *Peptoniphilus timonensis* sp. nov.

**DOI:** 10.4056/sigs.2956294

**Published:** 2012-09-26

**Authors:** Ajay Kumar Mishra, Jean-Christophe Lagier, Catherine Robert, Didier Raoult, Pierre-Edouard Fournier

**Affiliations:** 1 Unité de Recherche sur les Maladies Infectieuses et Tropicales Emergentes, UMR, Aix-Marseille Université, France

**Keywords:** *Peptoniphilus timonensis*, genome

## Abstract

*Peptoniphilus timonensis* strain JC401^T^ sp. nov. is the type strain of *P. timonensis* sp. nov., a new species within the *Peptoniphilus* genus. This strain, whose genome is described here, was isolated from the fecal flora of a healthy patient. *P. timonensis* is an obligate Gram-positive anaerobic coccus. Here we describe the features of this organism, together with the complete genome sequence and annotation. The 1,758,598 bp long genome (1 chromosome, no plasmid) contains 1,922 protein-coding and 22 RNA genes, including 5 rRNA genes.

## Introduction

*Peptoniphilus timonensis* strain JC401^T^ (= CSUR P165= DSM 25367) is the type strain of *P. timonensis* sp. nov. This bacterium is a Gram-positive, anaerobic, indole-positive coccus that was isolated from the stool of a healthy Senegalese patient as part of a “culturomics” study aiming at cultivating individually all species within human feces.

Since the early days of bacterial taxonomy, defining a bacterial species has been a matter of debate. Currently, the availability of a wide array of molecular methods, notably 16S rRNA and full genome sequencing, offers a possibility to base the description of new species on other methods than the “gold standard” of DNA-DNA hybridization [1]. In particular, sequence similarity of the 16S rRNA, although neither uniform across taxa nor necessarily predictive, enabled the taxonomic classification or reclassification of many taxa [2], and genome sequencing has provided access to the complete genetic information of bacteria [3]. As a consequence, we based our description of *P. timonensis* sp. nov. on a polyphasic approach [4] including their genome sequence and main phenotypic characteristics (habitat, Gram-stain reaction, culture and metabolic characteristics, MALDI-TOF spectrum, and when applicable, pathogenicity).

Here we present a summary classification and a set of features for *P. timonensis* sp. nov. strain JC401^T^ together with the description of the complete genomic sequencing and annotation. These characteristics support the creation of the *P. timonensis* species.

The genus *Peptoniphilus* (Ezaki *et al*. 2001) was created in 2001 [5] and consist of species that are non-saccharolytic, butyrate-producing, non-motile gram-positive anaerobic cocci and use peptones and oligopeptide as major energy source [6]. To date, the genus *Peptoniphilus* contains eight species namely *P. asaccharolyticus*, *P. harei*, *P. indolicus*, *P. ivorii*, *P. lacrimalis* [5], *P. gorbachii*, *P. olsenii* [6], *P. methioninivorax* [7]. Members of the genus *Peptoniphilus* have mostly been isolated from various human clinical specimens such as vaginal discharges, ovarian, peritoneal, sacral and lachrymal gland abscesses [5]. *P. indolicus* causes summer mastitis in cattle [5].

## Organism information

**Figure 1:**
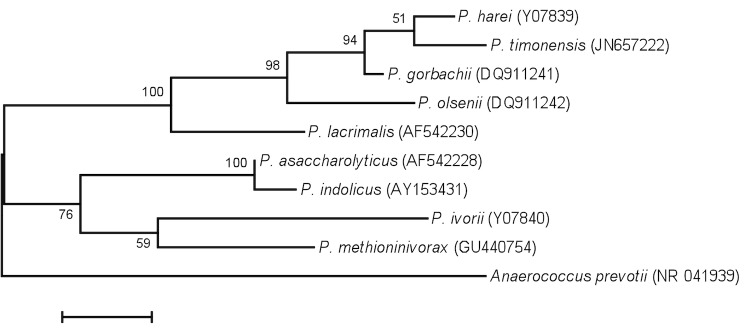
Phylogenetic tree highlighting the position of *Peptoniphilus timonensis* strain JC401^T^ relative to other type strains within the *Peptoniphilus* genus. GenBank accession numbers are indicated in parentheses. Sequences were aligned using CLUSTALW, and phylogenetic inferences obtained using the maximum-likelihood method within the MEGA software. Numbers at the nodes are percentages of bootstrap values obtained by repeating the analysis 500 times to generate a majority consensus tree. *Anaerococcus prevotii* was used as outgroup. The scale bar represents a 2% nucleotide sequence divergence.

A stool sample was collected from a healthy 16-year-old male Senegalese volunteer patient living in Dielmo (rural village in the Guinean-Sudanian zone in Senegal), who was included in a research protocol. The patient gave an informed and signed consent, and the agreement of the National Ethics Committee of Senegal and the local ethics committee of the IFR48 (Marseille, France) were obtained under agreement (09-022 and 11-017). The fecal specimen was preserved at -80°C after collection and sent to Marseille. Strain JC401^T^ was isolated in June 2011 by cultivation on 5% sheep blood-enriched Brain Heart Infusion agar (Becton Dickinson, Heidelberg, Germany). This strain exhibited a 98% nucleotide sequence similarity with *Peptoniphilus harei*, the phylogenetically closest validated *Peptoniphilus* species (Figure 1, for classification, see Table 1). This value was lower than the 98.7% 16S rRNA gene sequence threshold recommended by Stackebrandt and Ebers to delineate a new species without carrying out DNA-DNA hybridization [18].

**Table 1:** Classification and general features of *Peptoniphilus timonensis* strain JC401^T^ according to the MIGS recommendation [8]

**MIGS ID**	**Property**	**Term**	**Evidence code^a^**
		Domain *Bacteria*	TAS [9]
		Phylum *Firmicutes*	TAS [10-12]
		Class *Clostridia*	TAS [13,14]
	Current classification	Order *Clostridiales*	TAS [15,16]
		Family XI *Incertae sedis*	TAS [15,16]
		Genus *Peptoniphilus*	TAS [5]
		Species *Peptoniphilus timonensis*	IDA
		Type strain JC401^T^	IDA
	Gram stain	Positive	IDA
	Cell shape	Coccoid	IDA
	Motility	Nonmotile	IDA
	Sporulation	Nonsporulating	IDA
	Temperature range	Mesophile	IDA
	Optimum temperature	37°C	IDA
MIGS-6.3	Salinity	Growth in BHI medium + 1% NaCl	IDA
MIGS-22	Oxygen requirement	Anaerobic	IDA
	Carbon source	Unknown	NAS
	Energy source	Unknown	NAS
MIGS-6	Habitat	Human gut	IDA
MIGS-15	Biotic relationship	Free living	IDA
MIGS-14	Pathogenicity Biosafety level Isolation	Unknown 2 Human feces	NAS
MIGS-4	Geographic location	Senegal	IDA
MIGS-5	Sample collection time	September 2010	IDA
MIGS-4.1	Latitude -	13.7167	IDA
MIGS-4.1	Longitude	-16.4167	IDA
MIGS-4.3	Depth	Surface	IDA
MIGS-4.4	Altitude	51 m above sea level	IDA

Evidence codes - IDA: Inferred from Direct Assay; TAS: Traceable Author Statement (i.e., a direct report exists in the literature); NAS: Non-traceable Author Statement (i.e., not directly observed for the living, isolated sample, but based on a generally accepted property for the species, or anecdotal evidence). These evidence codes are from the Gene Ontology project [17]. If the evidence is IDA, then the property was directly observed for a live isolate by one of the authors or an expert mentioned in the acknowledgements.

**Figure 2:**
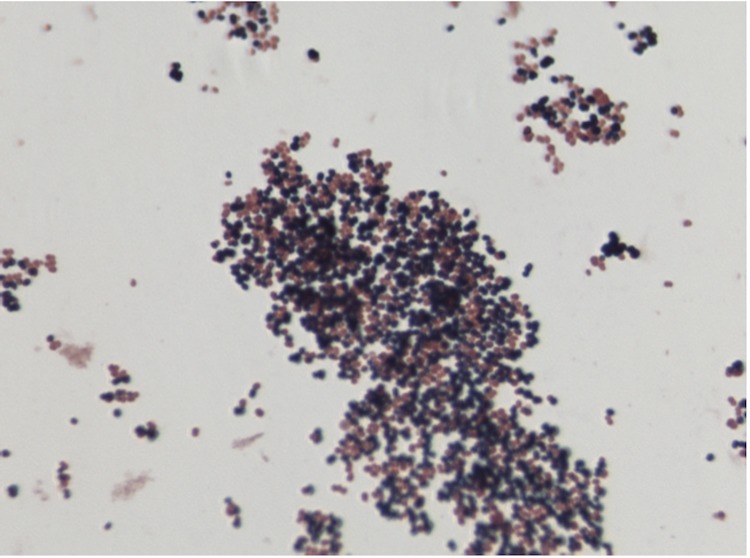
Gram staining of *P. timonensis* strain JC401^T^

**Figure 3:**
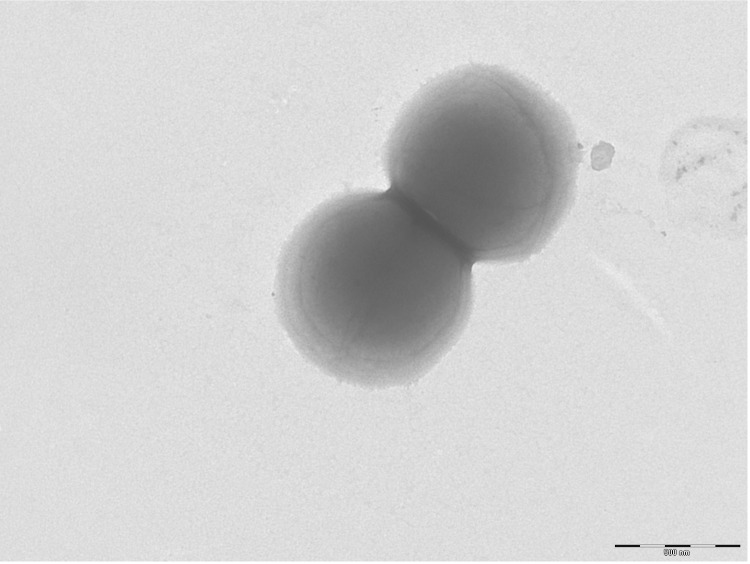
Transmission electron microscopy of *P. timonensis* strain JC401^T^, using a Morgani 268D (Philips) at an operating voltage of 60kV.The scale bar represents 900 nm.

Different growth temperatures (25, 30, 37, 45°C) were tested. Growth was not observed at 25°C and 45°C, but optimal growth occurred between 30 and 37°C. Colonies were 0.5 mm in diameter on blood-enriched BHI agar. Growth of the strain was tested under anaerobic and microaerophilic conditions using GENbag anaer and GENbag microaer systems, respectively (BioMérieux), and in aerobic conditions, with or without 5% CO_2_. Growth was not achieved in aerobic (with and without CO_2_) conditions. The growth was observed in anaerobic conditions. Gram staining showed Gram positive cocci (Figure 2). A motility test was negative. Cells grown on agar are sporulated and have a mean diameter of 0.91 µm (Figure 3).

Strain JC401^T^ exhibited a catalase activity but no oxidase activity. Using API Rapid ID 32A, positive reactions were obtained for α galactosidase, arginine arylimidase, tyrosine arylamidase, histidine arylamidase, serine arylamidase and indole production. Weak reactions were observed for leucine arylamidase and phenylalanine arylamidase. *P. timonensis* is susceptible to penicillin G, imipeneme, amoxicillin + clavulanic acid, vancomycin, clindamycin and metronidazole.

**Figure 4:**
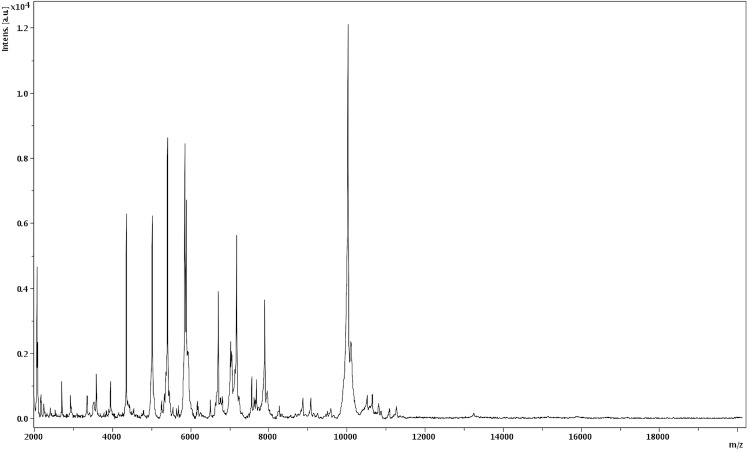
Reference mass spectrum from *P. timonensis* strain JC401^T^. Spectra from 12 individual colonies were compared and a reference spectrum was generated.

Matrix-assisted laser-desorption/ionization time-of-flight (MALDI-TOF) MS protein analysis was carried out as previously described [19]. Briefly, a pipette tip was used to pick one isolated bacterial colony from a culture agar plate, and to spread it as a thin film on an MTP 384 MALDI-TOF target plate (Bruker Daltonics, Leipzig, Germany). Twelve distinct deposits were done for strain JC401^T^ from twelve isolated colonies. Each smear was overlaid with 2µL of matrix solution (saturated solution of alpha-cyano-4-hydroxycinnamic acid) in 50% acetonitrile, 2.5% tri-fluoracetic-acid, and allowed to dry for five minutes. Measurements were performed with a Microflex spectrometer (Bruker). Spectra were recorded in the positive linear mode for the mass range of 2,000 to 20,000 Da (parameter settings: ion source 1 (IS1), 20 kV; IS2, 18.5 kV; lens, 7 kV). A spectrum was obtained after 675 shots at a variable laser power. The time of acquisition was between 30 seconds and 1 minute per spot. The twelve JC401^T^ spectra were imported into the MALDI BioTyper software (version 2.0, Bruker) and analyzed by standard pattern matching (with default parameter settings) against the main spectra of 3,769 bacteria, including 12 spectra from 8 *Peptoniphilus* species, which were used as reference data, in the BioTyper database. The method of identification included the m/z from 3,000 to 15,000 Da. For every spectrum, 100 peaks at most were taken into account and compared with spectra in the database. A score enabled the identification, or not, from the tested species: a score > 2 with a validated species enabled the identification at the species level, a score > 1.7 but < 2 enabled the identification at the genus level; and a score < 1.7 did not enable any identification. For strain JC401^T^, the obtained score was 1.2, thus suggesting that our isolate was not a member of a known species. We incremented our database with the spectrum from strain JC401^T^ (Figure 4). The spectrum was made available online in our free-access URMS database [20].

## Genome sequencing information

### Genome project history

The organism was selected for sequencing on the basis of its phylogenetic position and 16S rRNA similarity to other members of the *Peptoniphilus* genus, and is part of a “culturomics” study of the human digestive flora aiming at isolating all bacterial species within human feces. It was the seventh genome of a *Peptoniphilus* species and the first genome of *Peptoniphilus timonensis* sp. nov. The Genbank accession number is CAEL00000000 and consists of 97 large contigs. Table 2 shows the project information and its association with MIGS version 2.0 compliance.

**Table 2:** Project information

**MIGS ID**	**Property**	**Term**
MIGS-31	Finishing quality	High-quality draft
MIGS-28	Libraries used	One 454 paired end 3-kb library
MIGS-29	Sequencing platforms	454 GS FLX Titanium
MIGS-31.2	Fold coverage	35
MIGS-30	Assemblers	Newbler version 2.5.3
MIGS-32	Gene calling method	Prodigal
	Gold ID	Gi16876
	INSDC ID	PRJEB31
	NCBI project ID	CAEL00000000
	Genbank Date of Release	January 30, 2012
MIGS-13	Project relevance	Study of the human gut microbiome

### Growth conditions and DNA isolation

*P. timonensis* sp. nov. strain JC401^T^ (CSUR P165, DSM 25367) was grown anaerobically on 5% sheep blood-enriched Columbia agar at 37°C. Six petri dishes were spread and resuspended in 6x100µl of G2 buffer (EZ1 DNA Tissue kit, Qiagen). A first mechanical lysis was performed by glass powder on the Fastprep-24 device (Sample Preparation system, MP Biomedicals, USA) during 2x20 seconds. DNA was then treated with 2.5µg/µL lysozyme (30 minutes at 37°C) and extracted using the BioRobot EZ1 Advanced XL (Qiagen). The DNA was then concentrated and purified using the Qiamp kit (Qiagen). The yield and the concentration was measured by the Quant-it Picogreen kit (Invitrogen) on the Genios Tecan fluorometer at 123.3ng/µl.

### Genome sequencing and assembly

Five µg of DNA was mechanically fragmented on the Hydroshear device (Digilab, Holliston, MA,USA) with an enrichment size at 3-4kb. The DNA fragmentation was visualized through the Agilent 2100 BioAnalyzer on a DNA labchip 7500 with an optimal size of 2.47 kb. The library was constructed according to the 454 GS FLX Titanium paired end protocol. Circularization and nebulization were performed. After PCR amplification through 15 cycles followed by double size selection, the single stranded paired end library profile was visualized on an Agilent 2100 RNA Pico 6000 Labchip with an optimal at 568bp. Then the library was quantified on the Quant-it Ribogreen kit (Invitrogen) on the Genios_Tecan fluorometer at 890 pg/µL. The library concentration equivalence was calculated as 2.87E+09 molecules/µL. The library was stored at -20°C until further use.

The shotgun library was clonal amplified with 0.25 and 0.5cpb in 2 emPCR reactions per conditions with the GS Titanium SV emPCR Kit (Lib-L) v2.The yields of the emPCR were 2.79% and 10.79% respectively in the range of 5 to 20% from the Roche procedure.

Approximately 790,000 beads for a ¼ region and 340000 beads for a 1/8 region were loaded on the GS Titanium PicoTiterPlate PTP Kit 70×75 and sequenced with the GS FLX Titanium Sequencing Kit XLR70 (Roche). The run was performed overnight and then analyzed on the cluster through the gsRunBrowser and Newbler assembler (Roche). For the shotgun sequencing, 193,186 passed filter wells were obtained and generated 37.47Mb with a length average of 190 bp. The passed filter sequences were assembled Using Newbler with 90% identity and 40 bp as overlap. The final assembly identified 7 scaffolds and 97 large contigs (>1500bp) generating a genome size of 1.76 Mb

### Genome annotation

Open Reading Frames (ORFs) were predicted using Prodigal [21] with default parameters but the predicted ORFs were excluded if they were spanning a sequencing gap region. The predicted bacterial protein sequences were searched against the GenBank database [22] and the Clusters of Orthologous Groups (COG) databases using BLASTP. The tRNAScanSE tool [23] was used to find tRNA genes, whereas ribosomal RNAs were found by using RNAmmer [24] and BLASTn against the GenBank database. ORFans were identified if their BLASTP *E*-value was lower than 1e-03 for alignment length greater than 80 amino acids. If alignment lengths were smaller than 80 amino acids, we used an *E*-value of 1e-05. Such parameter thresholds have already been used in previous works to define ORFans.

To estimate the mean level of nucleotide sequence similarity at the genome level between *Peptoniphilus* species, we compared the ORFs only using BLASTN and the following parameters: query coverage of ≥ 70% and a minimum nucleotide length of 100 bp.

## Genome properties

**Figure 5:**
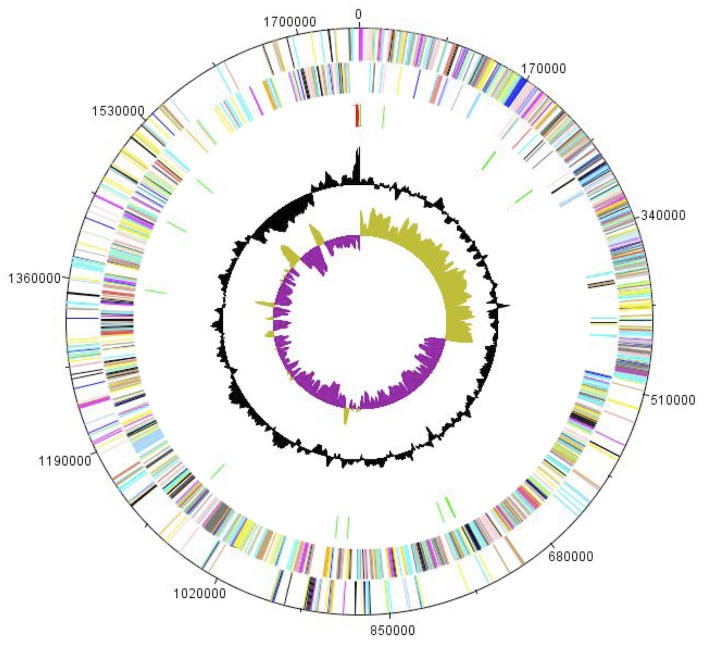
Graphical circular map of the chromosome. From outside to the center: Genes on the forward strand (colored by COG categories), genes on the reverse strand (colored by COG categories), RNA genes (tRNAs green, rRNAs red), GC content, and GC skew.

The genome is 1,758,598 bp long (1 chromosome, but no plasmid) with a 30.70% GC content (Figure 5 and Table 3). Of the 1,944 predicted genes, 1,922 were protein-coding genes and 22 were RNAs. A total of 1,368 genes (70.37%) were assigned a putative function. A total of 186 genes were identified as ORFans (9.6%). The remaining genes were annotated as hypothetical proteins. The distribution of genes into COGs functional categories is presented in Table 4. The properties and the statistics of the genome are summarized in Tables 3 and 4.

**Table 3:** Nucleotide content and gene count levels of the genome

Attribute	Value	% of total^a^
Genome size (bp)	1,758,598	
DNA coding region (bp)	1,566,468	89.07
DNA G+C content (bp)	5,398,89	30.7
Total genes	1,944	100
RNA genes	22	1.13
Protein-coding genes	1,922	98.87
Genes with function prediction	1,343	69.08
Genes assigned to COGs	1,368	70.37
Genes with peptide signals	119	6.12
Genes with transmembrane helices	450	23.15

^a^ The total is based on either the size of the genome in base pairs or the total number of protein coding genes in the annotated genome

**Table 4:** Number of genes associated with the 25 general COG functional categories

**Code**	**Value**	**%age**^a^	**Description**
J	140	7.28	Translation
A	0	0	RNA processing and modification
K	101	5.25	Transcription
L	128	6.66	Replication, recombination and repair
B	1	0.05	Chromatin structure and dynamics
D	24	1.25	Cell cycle control, mitosis and meiosis
Y	0	0	Nuclear structure
V	47	2.45	Defense mechanisms
T	62	3.23	Signal transduction mechanisms
M	66	3.43	Cell wall/membrane biogenesis
N	5	0.26	Cell motility
Z	0	0	Cytoskeleton
W	0	0	Extracellular structures
U	26	1.35	Intracellular trafficking and secretion
O	56	2.91	Posttranslational modification, protein turnover, chaperones
C	79	4.11	Energy production and conversion
G	40	2.08	Carbohydrate transport and metabolism
E	137	7.13	Amino acid transport and metabolism
F	61	3.17	Nucleotide transport and metabolism
H	58	3.02	Coenzyme transport and metabolism
I	41	2.13	Lipid transport and metabolism
P	77	4.01	Inorganic ion transport and metabolism
Q	15	0.78	Secondary metabolites biosynthesis, transport and catabolism
R	179	9.31	General function prediction only
S	133	6.92	Function unknown
-	554	28.82	Not in COGs

^a^ The total is based on the total number of protein coding genes in the annotated genome

## Comparison with the genomes from other *Peptoniphilus* species

Draft genome sequences are currently available for six species. Here we compared the genome sequence of *P. timonensis* strain JC401^T^ with those of *P. harei* strain ACS-146-V-Sch2b, *P. indolicus* strain ATCC BAA-1640 and *P. lacrimalis* strain 315-B.

The draft genome sequence of *P. timonensis* is larger than *P. lacrimalis* (1.76 Mb and 1.69 Mb, respectively) and smaller than *P. indolicus* and *P. harei* (2.2 Mb and 1.8 Mb, respectively). The G+C content of *P. timonensis* is comparable to *P. lacrimalis* (30.7 and 29.91% respectively) but smaller than those of *P. indolicus* and *P. harei* (32.29 and 34.44% respectively). Additionally, *P. timonensis* has more predicted genes than *P. harei* and *P. lacrimalis* (1,922, 1,724 and 1,589 respectively) and lesser than *P. indolicus* (2,269). The genes assigned to COGs of *P. timonensis* are comparable to *P. harei* (1,368 and 1,381 respectively) greater than *P. lacrimalis* (1,192) and lesser than *P. indolicus* (1,690). However, the distribution of genes into COG categories (Table 4) was almost similar in all the four genomes.

In addition, *P. timonensis* shared a mean 86.49% (range 77.75 to 99.15%), 85.54% (range 77.36 to 99.13) and 82.80% (range 77.43 to 95.39) sequence similarity with *P. harei*, *P. lacrimalis* and *P. indolicus* respectively at the genome level.

## Conclusion

On the basis of phenotypic, phylogenetic and genomic analyses, we formally propose the creation of *Peptoniphilus timonensis* sp. nov. that contains the strain JC401^T^. This strain has been found in Senegal.

### Description of *Peptoniphilus timonensis* sp. nov.

*Peptoniphilus timonensis* (tim.on.en’sis. L. gen. masc. n. *timonensis*, of Timone, the name of the hospital where strain JC401^T^ was cultivated. Isolated from stool from an asymptomatic Senegalese patient. *P. timonensis* is an anaerobic Gram-positive bacterium. Grows on axenic medium at 37°C in an anaerobic atmosphere. Strain JC401^T^ exhibited a catalase activity but no oxidase activity. Positive reactions were obtained for α galactosidase, arginine arylimidase, tyrosine arylamidase, histidine arylamidase, serine arylamidase and indole production. Weak reactions were observed for leucine arylamidase and phenylalanine arylamidase. Positive for indole. *P. timonensis* is susceptible to penicillin G, imipeneme, amoxicillin + clavulanic acid, vancomycin, clindamycin and metronidazole. Non-motile. The G+C content of the genome is 30.7%. The type strain is JC401^T^ (= CSUR P165= DSM 25367).
